# In Situ Calibration Method for an MGT Detection System Based on Helmholtz Coils

**DOI:** 10.3390/s26010191

**Published:** 2025-12-27

**Authors:** Ziqiang Yuan, Chen Wang, Yanzhang Xie, Yingzi Zhang, Wenyi Liu

**Affiliations:** 1 State Key Laboratory of Dynamic Measurement Technology, North University of China, Taiyuan 030051, China; 2 Precision and Intelligence Laboratory, Department of Measurement and Control Technology and Instruments, Chongqing University, Chongqing 400065, China

**Keywords:** Helmholtz coil, rapid calibration method, magnetometer array, magnetometer error

## Abstract

**Highlights:**

**What are the main findings?**

- Proposing an In Situ Calibration Method for Magnetic Gradient Tensor (MGT) Systems Based on Three-Axis Helmholtz Coils.
- A two-stage calibration process was established: Single-element nonlinear calibration and Array relative calibration.
- Closed-loop generation method for constant-magnitude random magnetic fields has been realized.

**What are the implications of the main findings?**

- Achieve stationary calibration without rotation, eliminating rotational errors and noise.
- Simultaneously compensates for magnetic sensor array scaling factors, non-orthogonality, soft iron/hard iron effects, and attitude inconsistencies.
- Significantly enhance the calibration stability and repeatability of magnetometer arrays.

**Abstract:**

Vector magnetometer arrays are essential for ferromagnetic target detection and MGT measurement, but their performance is limited by proportional factor errors, triaxial non-orthogonality, soft/hard iron interference, and inconsistent array orientations. Traditional rotation-based scalar calibration requires magnetic-free turntables or manual multi-orientation operations, introducing mechanical noise, orientation perturbations, and poor repeatability. This paper proposes an in situ rapid calibration method for MGT systems using triaxial Helmholtz coils. By generating three-dimensional magnetic field sequences of constant magnitude and random directions while keeping the sensors stationary, the method replaces conventional rotational excitation. A two-stage rapid calibration algorithm is developed to achieve individual sensor error modeling and array relative calibration. Experimental results show substantial improvements. The tensor invariant C_T_ decreased from 6287.84 nT/m to 7.57 nT/m, with variance reduced from 1.46 × 10^6^ to 13.47 nT^2^/m^2^; inter-sensor output differences were suppressed to 1–3 nT; and the magnetic field magnitude error dropped from ~940 nT to 3 × 10^−4^ nT, achieving a 5–6-order-of-magnitude enhancement. These results verify the method’s effectiveness in eliminating rotational errors, improving array consistency, and enabling high-precision in situ calibration with strong engineering value.

## 1. Introduction

Vector magnetometer arrays, capable of simultaneously measuring magnetic field magnitude and direction, find extensive applications in geological exploration, unexploded ordnance (UXO) detection, magnetic tracking of underwater vehicles, and space magnetic field measurement [1,2,3,4,5]. MGT measurement systems based on vector magnetometers can obtain more information about magnetic targets, thereby significantly improving target localization accuracy [6]. However, MGT arrays composed of multiple sensors are susceptible to various errors in practical applications, including scale factor errors, triaxial non-orthogonality errors, soft iron/hard iron interference, and inconsistent sensor mounting attitudes [7,8,9]. These errors not only disrupt the linearity of individual magnetometer outputs but also severely degrade response consistency among array elements, introducing systematic bias in gradient tensor calculations [10].

Current common magnetometer calibration methods primarily fall into two categories: vector calibration [11,12,13] and scalar calibration [14,15]. Vector calibration typically relies on precise attitude measurement equipment or standard magnetic field sources, involving high equipment costs and complex operations [16,17]. Scalar calibration involves rotating the magnetometer to collect magnetic field data at different orientations, then estimating parameters using the constraint that the geomagnetic field’s magnitude remains constant [18,19]. Although scalar methods are less costly and simpler to implement, they necessitate manual rotation or non-magnetic turntables to generate multi-attitude data. The rotation process inevitably introduces mechanical noise, acceleration disturbances, and human operational errors, leading to unstable calibration results with poor repeatability [20]. Furthermore, for MGT measurement systems, maintaining consistent rotation among multiple sensors simultaneously is more challenging, and attitude synchronization errors further reduce consistency between arrays [21].

Regarding the above issues, recent studies have explored replacing mechanical rotation with active magnetic field excitation methods, such as utilizing small-scale perturbation fields or combined fields from multiple fixed coils. However, simultaneously meeting the three requirements of “fixed orientation, diverse directions, and constant magnitude” remains challenging [22,23,24]. Since traditional ellipsoidal approximation methods are prone to bias in the presence of noise, generating sufficiently rich three-dimensional magnetic field directions under static conditions to achieve precise calibration remains an engineering challenge [25,26].

Beyond traditional vector and scalar approaches, researchers have also explored various optimization techniques and cross-domain methods to enhance calibration efficiency and accuracy. For instance, efficient calibration frameworks utilizing optimization algorithms have been successfully applied to multi-sensor systems, such as the fusion of cameras and laser rangefinders [27]. In the specific context of magnetic measurements, advanced solvers like the Simplex method have been investigated for the calibration of magnetoencephalography (MEG) devices [28]. Furthermore, the precise estimation of sensor position and orientation remains a critical challenge; recent studies have addressed automated estimation techniques in electromagnetic measurement facilities [29] and analyzed co-registration errors in optically pumped magnetometer (OPM) arrays [30]. These works collectively highlight the importance of robust optimization algorithms and precise geometric alignment in minimizing system errors.

To address this, this paper proposes an in situ rapid calibration method for MGT measurement systems based on triaxial Helmholtz coils. This method generates a three-dimensional magnetic field sequence with constant magnitude and randomly varying direction using triaxial Helmholtz coils while the array remains stationary, completely replacing traditional rotation operations and eliminating errors introduced by attitude perturbations at the source. Subsequently, a two-stage calibration process was implemented using the improved rapid calibration algorithm. First, nonlinear least-squares methods eliminate the proportionality factor, non-orthogonality, bias, and soft/hard iron effects of individual magnetometers. Second, least-squares estimation determines the relative pose transformation between arrays, unifying all sensor outputs to a common coordinate system and thereby enhancing the accuracy of MGT calculations.

The proposed method offers the following advantages:

(1) Stationary calibration eliminates the need for rotation, avoiding mechanical noise and significantly improving calibration stability and reproducibility.

(2) Comprehensive directional coverage of constant-magnitude random magnetic fields enhances parameter observability, leading to more accurate calibration.

(3) The two-stage calibration strategy simultaneously compensates for individual sensor errors and array attitude variations, achieving consistent array outputs.

(4) Experimental results demonstrate that this method significantly improves the measurement accuracy and consistency of the MGT measurement system, achieving improvements of 5–6 orders of magnitude in the tensor invariant C_T_, magnetometer output consistency, and magnetic field magnitude error.

In summary, this paper achieves high-precision in situ calibration of MGT measurement systems by constructing an active magnetic field excitation system under stationary conditions and integrating a rapid calibration algorithm, demonstrating high practical value and broad applicability in engineering applications.

## 2. Uniform Coil Magnetic Field and Rapid Calibration Method

### 2.1. Helmholtz Coils

#### 2.1.1. Field Strength Calculation Along the Helmholtz Coil Axis

A pair of equidistant coaxial coils forms a Helmholtz coil array, generating a highly uniform magnetic field in its central region. According to Biot–Savart law, the magnetic field at a distance z along the axis of a single square coil with side length L, N turns, and current I can be expressed by the standard model [31]:

(1) $$B(z)=\frac{4NIL^{2}}{\pi (L^{2}+4z^{2})\sqrt{2L^{2}+4z^{2}}}$$

When the inter-coil spacing is L/2, a square Helmholtz coil structure is formed. The combined magnetic field at the center after superposition of the two coils is:

(2) $$B_{HH}=2B(z=L/4)$$

This geometric configuration satisfies the Helmholtz condition, producing a highly uniform and approximately linear magnetic field distribution in the central region.

#### 2.1.2. Coupled Model of a Three-Axis Helmholtz System

Actual three-axis coil systems are not perfectly ideal. Geometric errors, mutual coupling effects, winding deviations, and non-orthogonal installation among the three coil sets result in a set of off-diagonal coupling relationships between the input current and the actual magnetic field [32,33]. To accurately describe the magnetic field output of the three-axis system, the following can be established:

(3) $$B=\left[\begin{array}{c} B_{x}\\ B_{y}\\ B_{z} \end{array}\right]=K\left[\begin{array}{c} I_{x}\\ I_{y}\\ I_{z} \end{array}\right]=KI$$

where matrix K is a 3 × 3 magnetic field coupling matrix:

(4) $$K=\left[\begin{array}{ccc} k_{xx} & k_{xy} & k_{xz}\\ k_{yx} & k_{yy} & k_{yz}\\ k_{zx} & k_{zy} & k_{zz} \end{array}\right]$$

The diagonal elements k_xx_, k_yy_, k_zz_ describe the magnetic field efficiency along the coil’s axis, while the off-diagonal elements k_xy_, k_xz_, ... reflect mutual coupling and non-orthogonality errors between different coils.

To achieve high-precision magnetic field control, this matrix requires calibration by solving:

(5) $$K=arg\;\underset{K}{\min}{\left\|B_{meas}-KI\right\|}^{2}$$

The estimated values of matrix K can be obtained using the least squares method.

The relationship between the three-axis magnetic field command B_d_ and the drive current is given by:

(6) $$I=K^{-1}B_{d}$$

This matrix compensation significantly reduces the effects of coil inter-axis coupling, winding asymmetry, and installation errors on the magnetic field direction and magnitude.

#### 2.1.3. Sources of Coil System Errors

To ensure the effectiveness of the constant-magnitude random magnetic field, it is necessary to specifically describe the sources of error in the coil system:

(1) Geometric errors: Deviations between actual coil dimensions and theoretical design.

(2) Current Drive Error: Limited power supply bandwidth causes slight delays in the dynamic magnetic field.

(3) Environmental magnetic noise: Includes geomagnetic disturbances and power-frequency magnetic fields generated by nearby equipment.

(4) Coil coupling error: Mutual inductance terms between the three-axis coils that are difficult to completely eliminate.

By employing a closed-loop control method (coil command → measured magnetic field → PI adjustment), the length error can be controlled to <0.5 nT, thereby meeting the high-precision requirements for magnetometer calibration.

#### 2.1.4. Calibration of the 3 × 3 Coupling Matrix K

During actual measurement, the Helmholtz coil initially operates in DC open-loop mode, where it functions independently of the triaxial fluxgate probe.

According to Section 2.1.2, to determine the K value, we positioned a three-axis fluxgate magnetometer (Model CH-370, integrated into the coil system; fluxgate sensor parameters are shown in the Table 1 and are superior to those of the fluxgate sensor to be calibrated) at the geometric center of the Helmholtz coil to serve as the reference sensor.

First, record magnetic field readings over a period of time while ensuring the current in all three-axis coils is zero (I = 0). Use the time-averaged value as the background magnetic field B_0_. Subsequently, sequentially apply N sets of different current vectors $I^{\left(n\right)}={\left[I_{x}^{\left(n\right)},I_{y}^{\left(n\right)},I_{z}^{\left(n\right)}\right]}^{T}(n=1,\ldots ,N)$ to the three-axis coils. This includes both positive and negative single-axis excitation for each axis, as well as several two-axis and three-axis combined excitations to fully activate all degrees of freedom in three-dimensional space. For each set of current vectors, the reference sensor records the corresponding measured magnetic field $B_{D}^{\left(n\right)}$. The differential magnetic field generated by the coil is calculated as

(7) $$B_{meas}^{\left(n\right)}=B_{D}^{\left(n\right)}-B_{0}$$

Stack all samples by column to form matrix:

(8) $$B_{meas}=\left[B_{meas}^{\left(1\right)},\ldots ,B_{meas}^{\left(N\right)}\right]\in R^{3\times N}$$

(9) $$I=\left[I^{\left(1\right)},\ldots ,I^{\left(N\right)}\right]\in R^{3\times N}$$

Ultimately, through least-squares fitting, the actual value of the coupling matrix K can be obtained as

(10) $$K=\left[\begin{array}{ccc} -41.222 & -0.153 & 0.250\\ -0.315 & 44.121 & -0.212\\ 0.211 & 0.139 & 39.708 \end{array}\right]$$

The measurement uncertainty of a magnetic field characterizes the dispersion of possible values for the true value of the measured magnetic field. When the coil operates in closed-loop mode, record the measured magnetic field $B_{M}^{\left(k\right)}$ corresponding to the three-axis magnetic field command $B_{d}^{\left(k\right)}$. For each test point, the relative error in the magnetic field modulus is calculated as

(11) $$\delta_{k}=\frac{\left\|B_{M}^{\left(k\right)}\right\|-\left\|B_{d}^{\left(k\right)}\right\|}{\left\|B_{d}^{\left(k\right)}\right\|}$$

Experimental results indicate that all $\left|\delta_{k}\right|$ values are confined within ±0.5%; thus, the relative expanded uncertainty of the magnetic field modulus at the coil center is better than 0.5%.

### 2.2. Cross-Shaped MGT Measurement System

MGT measurement systems can be categorized into various structural forms, including triangular, cross-shaped, square, and hexahedral configurations. Simulation comparisons of different structural forms indicate that the cross-shaped MGT measurement system offers consistent measurement points, minimal error, optimal structure, and a moderate number of sensors [34,35,36]. Therefore, this paper designs a cross-shaped MGT measurement system and performs error correction on it.

**Figure 1:**
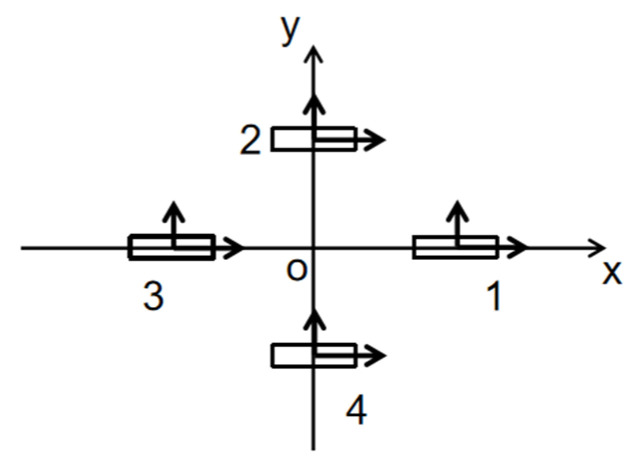
Schematic diagram of the cross-shaped MGT measurement system.

As shown in Figure 1, the cross-shaped magnetic field tensor detection system consists of four triaxial fluxgate vector magnetometers. The MGT represents the rate of change of the three components of the magnetic vector along three mutually orthogonal directions. Let the magnetic induction intensity at a point be B = [B_x_, B_y_, B_z_]^T^. Its magnetic field gradient tensor can be expressed as

(12) $$G=\left[\begin{array}{ccc} \frac{\partial B_{x}}{\partial x} & \frac{\partial B_{y}}{\partial y} & \frac{\partial B_{z}}{\partial z}\\ \frac{\partial B_{y}}{\partial x} & \frac{\partial B_{y}}{\partial y} & \frac{\partial B_{y}}{\partial z}\\ \frac{\partial B_{z}}{\partial x} & \frac{\partial B_{y}}{\partial y} & \frac{\partial B_{z}}{\partial z} \end{array}\right]=\left[\begin{array}{ccc} B_{xx} & B_{xy} & B_{xz}\\ B_{yx} & B_{yy} & B_{yz}\\ B_{zx} & B_{zy} & B_{zz} \end{array}\right]$$

The tensor invariant C_T_ is obtained by calculating the matrix norm of the tensor:

(13) $$C_{T}^{2}=\sum (G_{ij}{)}^{2}=(\frac{\partial B_{x}}{\partial x}{)}^{2}+(\frac{\partial B_{x}}{\partial y}{)}^{2}+(\frac{\partial B_{x}}{\partial z}{)}^{2}\;+(\frac{\partial B_{y}}{\partial x}{)}^{2}+(\frac{\partial B_{y}}{\partial y}{)}^{2}+(\frac{\partial B_{y}}{\partial z}{)}^{2}+(\frac{\partial B_{z}}{\partial x}{)}^{2}+(\frac{\partial B_{z}}{\partial y}{)}^{2}+(\frac{\partial B_{z}}{\partial z}{)}^{2}\;C_{T}=\sqrt{C_{T}^{2}}=k\frac{\mu_{0}}{4\pi }\frac{M}{r^{4}}$$

The more consistent the calibrated sensor outputs, the more stable the gradient tensor invariant becomes. This quantity serves as a crucial metric for subsequent system performance evaluation.

The choice of the tensor invariant C_T_ as the primary evaluation metric for magnetic gradient tensor errors is based on two main reasons:

(1) The magnetic gradient tensor comprises nine components (including five independent components). Under the combined influence of various error sources such as scale factor errors, attitude errors, and baseline errors, the magnitude and trend of deviations across different components are inconsistent. Observing only one or a few components typically yields only partial information, making it difficult to comprehensively reflect the overall error level of the system. The C_T_ is composed of all independent components, serving as a comprehensive measure of the total error energy within the tensor. Deviations in any single component accumulate within the C_T_, making it a more suitable metric for evaluating overall system accuracy.

(2) C_T_ is a tensor invariant, whose value remains unchanged under any orthogonal coordinate rotation. For magnetic gradient tensor systems operating in different orientations or reference coordinate systems, the overall error metric should not depend on the specific coordinate system chosen. By employing C_T_ as a rotation-invariant scalar, results from different experimental conditions and orientation configurations can be made comparable, making it a more suitable primary indicator for overall system performance.

Additionally, typical sensor errors such as scale factors, bias, attitude, and baseline will introduce non-zero deviations in one or more gradient components. Since C_T_ is a nonlinear function composed of entirely independent components, anomalies in any component cause C_T_ to deviate from zero. Consequently, C_T_ exhibits global sensitivity to various sensor error types, making it suitable as a representative measure of overall error energy.

### 2.3. Rapid Calibration Method

This study proposes a two-stage rapid calibration process designed to efficiently calibrate magnetometer arrays. This process first employs a monolithic calibration stage to independently correct the internal errors of each magnetometer. Subsequently, relative array calibration unifies the outputs of all magnetometers to a common reference coordinate system. The process innovatively utilizes Helmholtz coils to generate a constant-magnitude random magnetic field, enabling sufficient parameter observability without relying on physical rotation during calibration.

#### 2.3.1. Individual Calibration (Constant-Magnitude Random Magnetic Field Constraint)

The magnetometer output model is:

(14) $$B_{m}=AH_{e}+b+n$$

where A: matrix containing proportionality factor and non-orthogonal error, b: hard-iron bias, n: noise term

The ideal magnetic field magnitude satisfies:

(15) $$\left\|H\right\|=H_{0}$$

Therefore:

(16) $$A^{-1}\left(B_{n}-b\right)=H_{n}$$

This study employs the Levenberg–Marquardt (LM) algorithm to estimate A^−1^ and b. Compared to traditional ellipsoidal fitting methods, this approach utilizes a constant-magnitude magnetic field instead of the geomagnetic field, thereby avoiding geomagnetic drift. The random direction coverage of the spherical surface generated by Helmholtz coils ensures good parameter observability without requiring rotation, thus eliminating attitude and acceleration noise. After monolithic calibration, the true output of each magnetometer can be obtained:

(17) $$H=A^{-1}\left(B_{n}-b\right)$$

**Figure 2:**
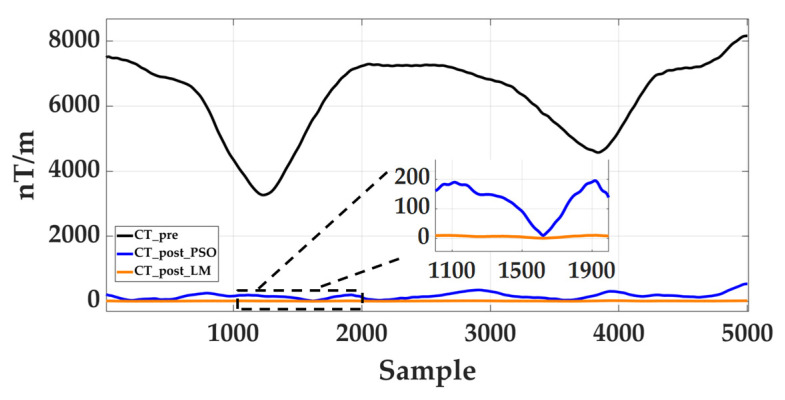
Comparison of PSO algorithm and LM algorithm before and after correction.

In addition to the LM algorithm employed in this paper, calibration problems can also be solved using stochastic global optimization or machine learning (ML)-assisted methods. For instance, stochastic methods such as Particle Swarm Optimization (PSO), Genetic Algorithm (GA), and Simulated Annealing (SA) do not rely on gradient information, are insensitive to initial conditions, and are theoretically applicable to multi-modal or strongly non-convex objective functions. To validate the applicability of stochastic optimization methods in this study, we employed PSO to solve for the ellipsoidal calibration parameters of the reference sensor under identical scalar-constrained objective functions and datasets as LM. The obtained calibration parameters were then applied to array uniformization and cross-difference gradient reconstruction. The final evaluation was conducted using the tensor invariant C_T_ as a comprehensive consistency metric. As shown in Figure 2, the PSO-corrected C_T_ exhibits a mean of 160.12 nT/m and variance of 8500.25 nT^2^/m^2^. In contrast, the LM-corrected C_T_ yields a mean of 7.57 nT/m and variance of 13.47 nT^2^/m^2^, demonstrating significantly lower overall error levels and higher stability. This comparison demonstrates that for the calibration problem addressed in this paper, LM converges more efficiently and stably to optimal solutions, making it the preferred default solver. In contrast, stochastic methods like PSO tend to remain in suboptimal regions without detailed hyperparameter tuning or local refinement, resulting in higher residual levels in the corrected C_T_.

Additionally, ML-assisted methods can serve as alternatives, such as training regression models or constructing surrogate models to accelerate calibration solutions. However, ML-assisted methods typically exhibit “black-box” characteristics with limited interpretability, sensitivity to training data coverage and distribution consistency, and potential issues with cross-environment generalization. Given this paper’s objective to provide a reproducible, interpretable, and easily engineered in situ calibration workflow, we prioritize LM as the deterministic solver in this study. Future work will further explore the potential advantages of ML-assisted calibration strategies in complex error models and online adaptive scenarios.

#### 2.3.2. Array Relative Calibration (Unified Coordinate System)

Due to installation orientation deviations, a linear transformation relationship must be established between each magnetometer and the reference magnetometer:

(18) $$H_{i}=T_{i}H_{ref}+t_{i}$$

where T_i_ contains nine rotation-related parameters, and t_i_ represents the offset difference.

After linearization, three equations can be generated for each sampling point. Solving these equations after stacking N samples yields:

(19) $$\theta =arg\;\underset{\theta }{\min}{\left\|A\theta -y\right\|\;}^{\;2}$$

When N ≥ 4, the solution can be obtained via the least squares method:

(20) $$[T_{i},t_{i}]=(A^{T}A{)}^{-1}A^{T}y$$

This step unifies all magnetometers into a common coordinate system, ensuring consistency for subsequent gradient tensor calculations.

#### 2.3.3. Final Output of Consistency

Ultimately, the overall calibration output for the i-th magnetometer is:

(21) $$H_{i}^{final}=T_{i}A_{i}^{-1}(B_{i}-b_{i})+t_{i}$$

At this point, the proportional factor, bias, non-orthogonality error, soft iron/hard iron effects, and attitude inconsistencies within the array are fully compensated.

First, Equation (18) converts the measurement error of each magnetometer into an error relative to the reference magnetometer. This approach involves calibrating a reference magnetometer first and then aligning the magnetometers in the other gradient arrays to it. For the calibration process of a cross-shaped MGT array, the proposed calibration method becomes more efficient, thus offering unique advantages over existing procedures.

## 3. MGT Measurement System for Helmholtz Coils

**Figure 3:**
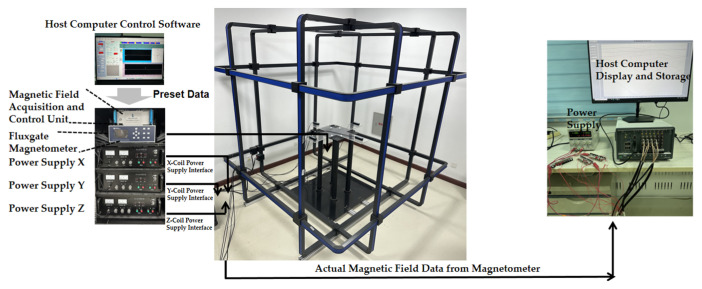
Schematic of the Helmholtz coil in situ calibration system.

This study established a three-dimensional Helmholtz coil system for in situ calibration of magnetometer arrays, comprising a magnetic field generator, a measurement and control unit, and a magnetic field detection unit, as shown in Figure 3. The system utilizes a mobile control terminal, magnetometers, a magnetic field acquisition control power supply, and three power amplifiers. The three power amplifiers are powered by 220 V AC. Their output interfaces are connected to the X, Y, and Z terminals of the 3D coil, respectively. The communication interface of the magnetic field control unit connects to the mobile control terminal via a network cable, into which fixed magnetic field magnitude data is written. A non-magnetic platform with the magnetometer securely mounted is positioned at the center of the uniform magnetic field generated by the three-dimensional coil for data acquisition. The collected data undergoes rapid calibration methods, ultimately achieving a three-dimensional magnetic field sequence with constant magnitude and randomly varying direction while the array remains completely stationary. This enables rapid, high-precision in situ calibration of the magnetometer array.

### 3.1. Introduction to Helmholtz Coils

**Figure 4:**
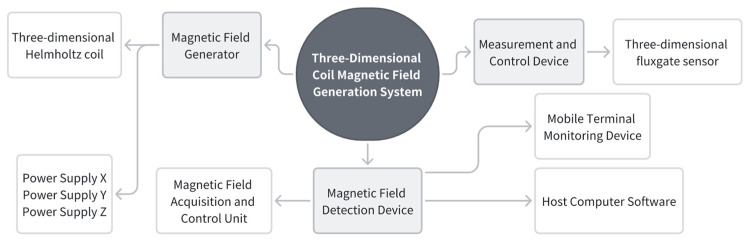
Helmholtz coil system architecture.

The high-dynamic geomagnetic shielding system in this project comprises a magnetic field generator, a measurement and control unit, and a magnetic field detection device, as shown in Figure 4.

Helmholtz coils are formed by connecting two coils with identical radii and turns, arranged coaxially with a spacing equal to their radius. They can generate magnetic fields ranging from extremely weak to hundreds of gauss (depending on coil turns, dimensions, and field strength). A three-dimensional Helmholtz coil system is formed by assembling three such coil sets orthogonally in space. It can generate magnetic fields in the X, Y, and Z directions within a Cartesian coordinate system, or synthesize two-dimensional or three-dimensional vector magnetic fields in any desired direction. Each coil set is equipped with an independent power supply to drive constant or dynamic working currents in the corresponding direction. The magnetic field detection device is a three-dimensional fluxgate vector magnetometer probe. The magnetometer acquires magnetic field signals, which are subsequently amplified, filtered, and processed through analog-to-digital conversion. The magnetic field information is finally displayed on the screen and can be uploaded via the RS232 serial port. The measurement and control system comprises a mobile terminal control device, a magnetic field acquisition control unit, and host computer software. The host computer communicates with the magnetic field acquisition control unit via Ethernet to set target magnetic fields for each axis. Simultaneously, it reads real-time magnetic field data returned by the magnetometer through the serial port, establishing a closed-loop control system of "magnetic field setting-detection-adjustment." This system employs a PI algorithm to achieve high-precision regulation of the internal magnetic field within the coils.

To ensure constant magnitude of random magnetic fields throughout calibration, the system employs closed-loop control. This strategy first generates target magnetic field B_d_, which is converted into drive current I and applied to the three-axis coils via a power amplifier. Simultaneously, the reference magnetometer continuously measures the actual spatial magnetic field B_m_. The PI controller dynamically feeds back the magnetic field error $∆B=B_{d}-B_{m}$ to the current regulation stage until the actual field satisfies $\left\|B\right\|=H_{0}\pm 0.5\;nT$. This closed-loop mechanism significantly enhances the stability of the random magnetic field sequence, providing reliable input for individual element calibration.

### 3.2. Introduction to the Cross-Type Magnetometer Measurement System

**Figure 5:**
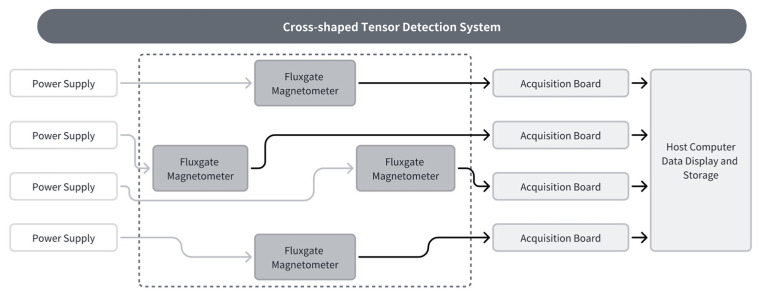
Cross-type magnetometer measurement system architecture.

The cross-shaped magnetometer measurement system comprises a non-magnetic platform and four triaxial magnetometer sensors, each with a baseline length of 400 mm, as shown in Figure 5. 

Synchronized data acquisition for all four sensors is achieved via the PXIe-4464 dynamic signal acquisition module. In this system, the power supply feeds the fluxgate magnetometers, which handle signal acquisition. These convert magnetic signals into voltage signals transmitted to the corresponding PXIe-4464 acquisition board (NIPXIe-4464 is a high-precision dynamic signal acquisition module featuring four inputs and two outputs, 24-bit resolution, a sampling rate of 204.8 kS/s per channel, and integrated IEPE signal conditioning). It performs IEPE conditioning and ADC conversion before uploading data to the host computer, which handles data display and storage. This system enables synchronous sampling from four magnetometers, ensuring subsequent gradient tensor calculations remain unaffected by sampling timing discrepancies.

### 3.3. Preset Data Generation

To obtain highly observable calibration data, this study designed a random magnetic field sequence satisfying the following constraints. A set of three-dimensional vector sequences meeting the constant magnitude $\left\|B\right\|$ = 86602 nT was generated using MATLAB(R2023a) (i.e., when B_x_ = 50000 nT, B_y_ = 50000 nT, B_z_ = 50000 nT). First, several directions (θ, φ) were randomly selected or specified in spherical coordinates, converted to unit vectors $\hat{u}=(sin\theta cos\phi ,sin\theta sin\phi ,cos\theta )$, and then each unit vector was multiplied by the scalar 86602 to obtain the target field vector B = (B_x_, B_y_, B_z_) = 86602∙$\hat{u}$, ensuring constant magnitude for each data set. Subsequently, these target magnetic field values are converted into corresponding drive current sequences via a pre-measured coil coupling matrix. Low-pass filtering and rate limiting are performed in MATLAB(R2023a) to meet coil bandwidth and power supply constraints. Finally, the current sequences are transmitted to the three-axis Helmholtz coils via a data acquisition card. Real-time measurements from reference magnetometers guide current fine-tuning, suppressing non-ideal coupling and environmental interference to <±0.5 nT. The resulting random constant-magnitude magnetic field sequences exhibit uniform directional distribution, stable magnitude, and low noise, making them suitable as input data for subsequent calibration.

### 3.4. Magnetometer Array Calibration Process

**Figure 6:**
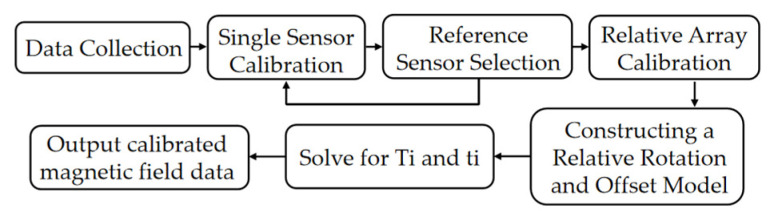
Magnetometer array calibration process.

To eliminate proportional factor errors, non-orthogonality errors, soft iron interference, and hard iron interference arising during magnetometer manufacturing and installation, while achieving coordinate uniformity among array members, this study proposes a phased calibration algorithm. This algorithm comprises individual magnetometer calibration and array relative calibration stages, culminating in overall consistency output. The complete workflow is illustrated in Figure 6:

(1) Individual Magnetometer Calibration

The magnetometer acquires a random constant-magnetic-field sequence in a stationary state. The LM algorithm is employed to estimate parameters such as the proportional factor, non-orthogonality error, and soft/hard iron bias.

(2) Array Relative Calibration

Under identical magnetic field sequences, the calibrated outputs from each magnetometer should describe the same true magnetic field. The relative rotation and bias matrices are estimated using the least squares method.

(3) Global Consistent Output

All sensors are unified to a reference coordinate system, ultimately outputting highly consistent magnetic field data for gradient tensor computation.

This workflow achieves true “in-situ calibration” without requiring rotation or altering array orientation, significantly enhancing calibration efficiency and stability.

## 4. Magnetometer Array Calibration Experiment

**Figure 7:**
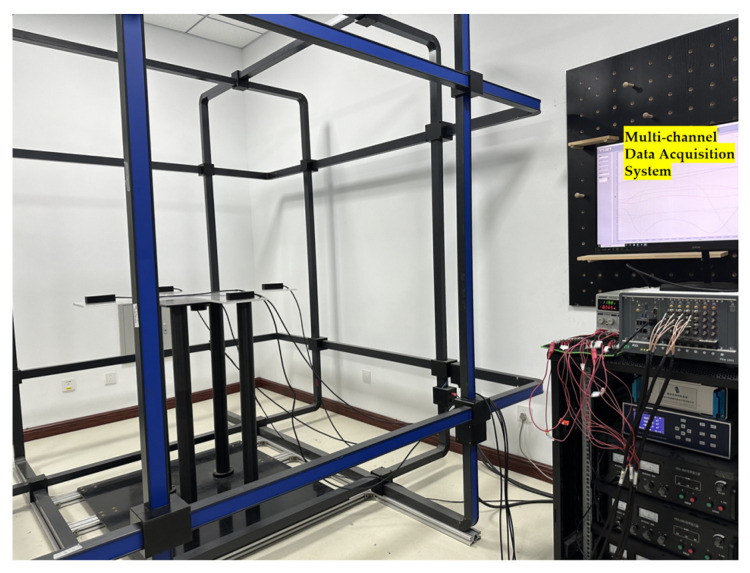
In situ calibration experiment.

To validate the proposed in situ rapid calibration method based on Helmholtz coils, an experimental platform, as shown in Figure 7, was constructed. The magnetometer array was placed at the center of the uniform magnetic field generated by a three-axis Helmholtz coil, and calibration data were acquired using a predefined random constant-magnitude magnetic field sequence. Subsequently, the MGT invariants, consistency metrics, and magnetic field magnitude errors before and after calibration were compared to evaluate the method’s performance.

### 4.1. Experimental Platform and Data Acquisition

The magnetometer array comprises four triaxial fluxgate magnetometers mounted at the vertices of a cross configuration. The baseline lengths for the X and Y axes are 400 mm and 400 mm, respectively.

During the experimental setup and measurement process, we specifically considered the impact of electromagnetic compatibility (EMC) and background magnetic field noise on calibration results. To minimize external electromagnetic interference, all Helmholtz coil calibration experiments were scheduled during late-night hours. On one hand, the geomagnetic field exhibits slower variations at this time, resulting in minimal time-varying components in the background magnetic field. On the other hand, large electrical equipment within the building (such as air conditioning units and elevators) typically operates at low load or is shut down during these hours. The absence of high-power transformers and high-current busbars in the vicinity significantly suppresses additional magnetic field interference introduced by the mains power supply and electrical equipment.

Before each calibration begins, keep the three-dimensional Helmholtz coil de-energized. The built-in reference triaxial magnetometer (CH-370, superior to Mag690-FL100, with specific parameters as shown in Table 1) acquires a background magnetic field time series while the array remains de-energized, then transmits the data to the host computer. The host computer first estimates the three-axis static background field components based on this data. It then controls the coil to generate an equal-amplitude, opposite-direction compensation field, maximally canceling the static geomagnetic field at the coil’s center. On this basis, the three-axis command for the target uniform magnetic field is superimposed to achieve precise generation of the target field.

**Figure 8:**
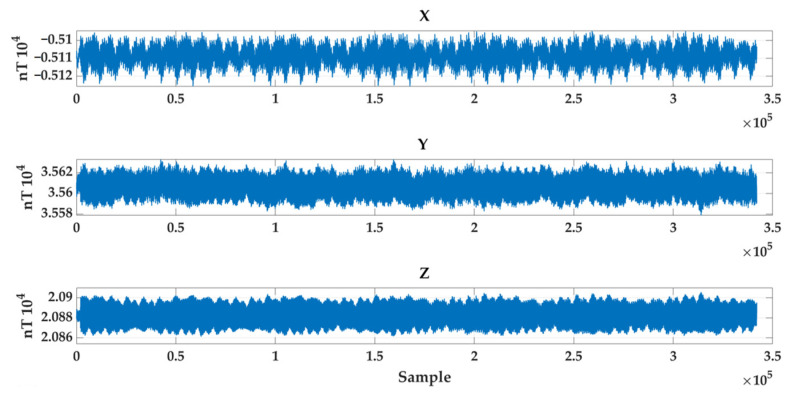
Nighttime background magnetic field.

To quantify the impact of background noise relative to the coil magnetic field, we collected nocturnal background magnetic field data using the CH-370 magnetometer. Its mean value and fluctuation range are quantified as shown in Figure 8, with the quantitative data presented in Table 2. The nocturnal background magnetic field exhibits minimal fluctuation, and its residual variation amplitude relative to the calibration field generated by the Helmholtz coils can be considered negligible. This demonstrates that under the calibration experimental conditions presented in this work, environmental electromagnetic interference and geomagnetic noise will not exert a dominant influence on subsequent magnetic gradient tensor correction results.

The parameters of the Mag690-FL100 magnetometer used in the experiment are listed in Table 3. The magnetometer array was placed at the center of a uniform magnetic field generated by a Helmholtz coil. By driving the current in the three-axis coils with preset data, a three-dimensional magnetic field distribution with constant magnitude and randomly varying direction was generated, enabling the magnetometer array to complete magnetic field sampling in a stationary state.

### 4.2. Improvement of MGT Invariant C_T_

**Figure 9:**
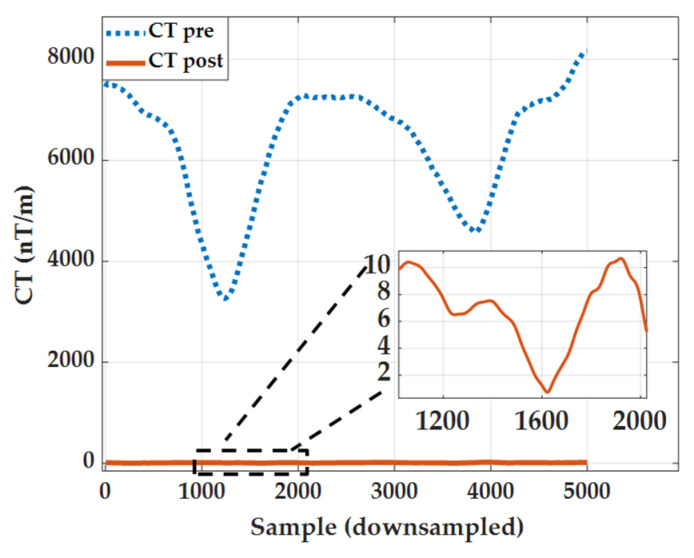
Comparison of tensor invariants before and after correction.

The MGT invariant C_T_ serves as a crucial metric for evaluating array errors. If the array remains uncalibrated, inconsistent directions and amplitudes in magnetometer outputs will generate numerous spurious gradients in the gradient tensor, causing C_T_ to deviate significantly from its true value. Figure 9 visually illustrates the temporal variation of the tensor invariant C_T_ before and after calibration. Prior to calibration, C_T_ deviates significantly from zero with pronounced fluctuations, whereas post-calibration it is largely compressed near zero, indicating substantial suppression of overall array tensor error. Table 4 quantitatively presents the changes in the tensor invariant C_T_ before and after correction. Before correction, the mean C_T_ value was 6287.84 nT/m, indicating a significant systematic tensor bias in the uncalibrated array. The variance of C_T_ was 1.46 × 10^6^ nT^2^/m^2^, reflecting intense fluctuations in the tensor invariant during sampling and highly unstable array output. After correction, the mean C_T_ value decreased to 7.57 nT/m—approximately 830 times lower (99.88%) than pre-correction—indicating near-complete elimination of overall bias. Residual tensor invariants approached zero, restoring the array response to near-ideal linear field structure. The variance of C_T_ decreased from 1.46 × 10^6^ nT^2^/m^2^ to 13.47 nT^2^/m^2^, indicating that the random fluctuations of C_T_ were also greatly suppressed. The stability of tensor measurements significantly improved, with the mean and variance decreasing by approximately three to five orders of magnitude, respectively. The correction nearly completely eliminates systematic tensor errors, suppressing the originally highly fluctuating C_T_ directly to near zero, demonstrating that the array response has fully recovered its linear spatial structure.

### 4.3. Evaluation of Internal Consistency in Array Output

To validate the calibration effect from the perspective of output consistency within the array, this paper employs the inter-sensor output difference:

(22) $$d_{i}=\left\|B_{i}^{final}-H_{i}^{final}\right\|$$

**Figure 10:**
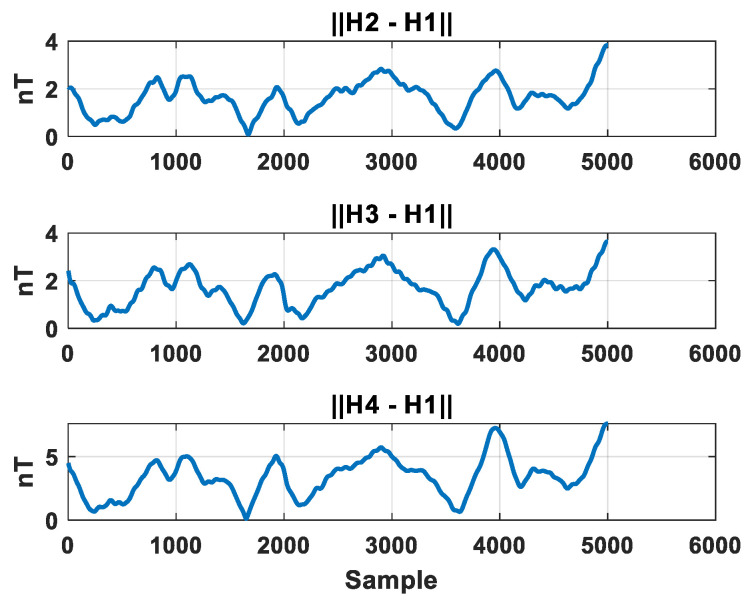
Sensor output difference fluctuations.

As a consistency metric, it measures the deviation among magnetometers when measuring the same true magnetic field. Figure 10 illustrates the fluctuation of output differences between each magnetometer and the reference magnetometer. After calibration, the three difference curves nearly overlap and fluctuate slightly around zero, indicating highly consistent outputs within the array. Table 5 quantifies the internal consistency of the array in terms of mean and variance. After calibration, the difference between each channel and the reference channel is only 1–3 nT, with variance not exceeding approximately 2.4 nT^2^. The outputs of the four sensors are nearly identical after calibration, indicating that the array has achieved the output characteristics of a “single equivalent magnetometer”. This is a prerequisite for subsequent high-precision gradient tensor calculations.

### 4.4. Effect of Magnetic Field Magnitude Error Suppression

**Figure 11:**
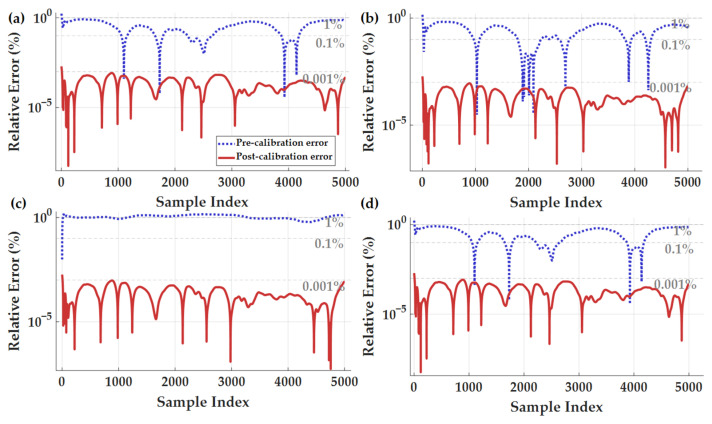
Comparison of relative error in magnetic field magnitude before and after correction: (**a**) sensor 1, (**b**) sensor 2, (**c**) sensor 3, (**d**) sensor 4.

Magnetic field magnitude error reflects the linearization degree of individual magnetometers and the overall scaling consistency of the array. Therefore, this paper adopts the relative magnitude error as another consistency metric. Magnitude error is a key indicator for assessing the linearity of a magnetometer. Ideally, the calibrated output should equal Hm. Figure 11 illustrates the variation in relative magnitude error for each sensor before and after calibration under random constant-mode excitation. The formula for calculating relative magnitude error is as follows:

(23) $$Relative\;Error\;B\left(\%\right)=\frac{\left|B_{i}^{final}-H_{m}\right|}{H_{m}}$$

(24) $$Relative\;Error\;H\left(\%\right)=\frac{\left|H_{i}^{final}-H_{m}\right|}{H_{m}}$$

Prior to calibration, the relative error approached 1%, indicating significant proportionality and offset errors in the raw outputs. After calibration, the curves consistently fell below 0.001%, demonstrating that systematic nonlinearity and scaling errors have been largely eliminated. Table 6 quantitatively demonstrates the performance difference between the four sensors before and after calibration, based on the mean and variance of the magnitude error. The pre-calibration error reached 940 nT with substantial fluctuation, indicating severe magnitude drift. Post-calibration, the mean magnitude error was compressed by 5–6 orders of magnitude, and the variance decreased by approximately 10^5^ orders of magnitude. The residual magnitude error is only about 0.3 nT, indicating that the proposed in situ calibration method can linearize the array output to the sub-nT level under static conditions, compressing the magnitude error to near the instrument noise floor (10^−4^ nT level). After calibration, the magnitudes of the four sensors are highly consistent, closely matching the theoretical values, with proportional factor, soft iron, hard iron, and rotation errors almost completely suppressed.

The results demonstrate that the proposed calibration method effectively eliminates proportional factor errors, bias, soft iron/hard iron effects, and sensor installation inconsistencies, achieving highly uniform array outputs and providing a reliable foundation for high-precision MGT measurements.

### 4.5. Comparison to Traditional Rotational Calibration

**Figure 12:**
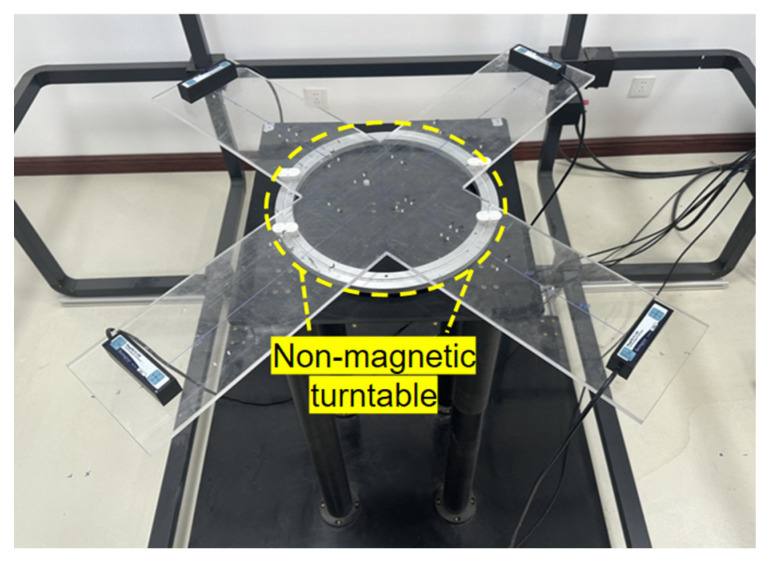
Schematic Diagram of Traditional Method Operation.

To ensure fair comparison, traditional scalar calibration experiments were conducted using the same magnetometer array under strictly controlled identical conditions. The specific procedure is as follows. First, the sensor array was mounted on a non-magnetic cross-shaped structure, which was then placed on a non-magnetic turntable as shown in Figure 12. Magnetic field data were collected at different orientations using traditional manual multi-posture operations. Second, calibration parameters—including scale factor, offset, and non-orthogonality—were estimated using a standard ellipsoid fitting algorithm [18]. Finally, to ensure validity, experiments were conducted in a low-magnetic-noise environment to minimize external interference, confining primary error sources to alignment errors caused by rotation and mechanical jitter.

**Figure 13:**
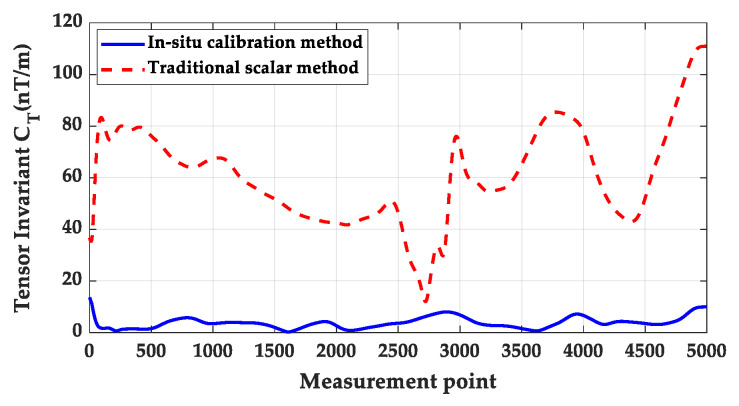
Tensor-invariant C_T_ calibrated using the method described herein and the conventional rotational calibration method.

Figure 13 compares the tensor-invariant C_T_ values obtained from both methods for system measurement calibration. Results indicate that the C_T_ values calibrated via traditional manual multi-pose operations are significantly higher than those from the proposed method, exhibiting greater fluctuation. This substantial error highlights the introduction of human error and attitude disturbance in the traditional rotational calibration method. Experimental results are summarized in Table 7, where data confirms that the proposed method delivers more precise calibration outcomes.

## 5. Conclusions and Discussion

This paper addresses the issues of large rotational errors, low efficiency, and poor repeatability in traditional calibration methods for MGT detection systems. It proposes an in situ rapid calibration method based on a three-axis Helmholtz coil. By generating a three-dimensional magnetic field sequence with constant magnitude and randomly varying direction while the array remains stationary, combined with a two-stage calibration strategy comprising nonlinear and linear least-squares methods, this approach achieves individual coil error compensation and array coordinate unification. Experimental results demonstrate that this method significantly enhances the accuracy and consistency of the MGT measurement system; the mean and variance of the tensor invariant C_T_ decrease by approximately five orders of magnitude, the magnitude error reduces from nearly 940 nT to 3 × 10^−4^ nT, and the sensor output variation within the array is compressed to 1–3 nT. These results validate the method’s effectiveness in suppressing systematic errors and improving array consistency and stability. This method requires no rotation, can be implemented on-site at low cost, and is suitable for engineering applications such as underwater navigation, UXO detection, and aerial magnetic surveys.

Despite its strong performance, three areas warrant further investigation: (1) extending the method to more complex array configurations like three-dimensional hexahedrons; (2) refining coil geometry and coupling error compensation to enhance ultimate accuracy; (3) optimizing random magnetic field generation strategies and error suppression in dynamic scenarios to improve observability and environmental adaptability. In summary, the proposed in situ rapid calibration method provides a stable, effective, and easily deployable calibration solution for high-precision MGT measurement systems, laying the foundation for future extension to more complex arrays and dynamic application scenarios.

## Figures and Tables

**Table 1:** CH-370 parameter specifications.

Accuracy Specifications	Parameter Value
Range	±100 µT
Zero-field offset error	±5 nT
Resolution	0.01 nT
Accuracy	<0.2% ± 5 nT of the reading
Sensor Noise	6pTRMS/√Hz at 1Hz
Three-dimensional orthogonality error	<0.1°

**Table 2:** Quantitative data of nighttime background magnetic fields.

Indicator	X-Axis	Y-Axis	Z-Axis
Mean Value (nT)	−5108.9	35,606.6	20,882.5
Variance (nT^2^)	27.31	72.85	72.78

**Table 3:** Mag690-FL100 parameter specifications.

Accuracy Specifications	Parameter Value
Background Noise	≤25pTRMS/√Hz@1 Hz
Linearity Error	0.01% F.S.
Orthogonality error	<1°
Offset error	±100 nT
Offset Temperature Drift	±2 nT/°C
Calibration error	±1%

**Table 4:** Comparison of tensor invariant C_T_ before and after correction.

Indicator	Before Correction	After Correction	Change Rate
C_T_ Mean Value (nT/m)	6287.84	7.57	−99.88%
C_T_ Variance (nT^2^/m^2^)	1,458,973.59	13.47	−99.999%

**Table 5:** Consistency of calibrated magnetometer array.

Indicator	||H2-H1||	||H3-H1||	||H4-H1||
Mean (nT)	1.6360	1.6865	3.3938
Variance (nT^2^)	0.4758	0.5842	2.3740

**Table 6:** Comparison of relative error in magnetic field magnitude before and after calibration.

Sensor	||Bi-Hm||Mean (nT)	||Hi-Hm||Mean (nT)	||Bi-Hm||Variance (nT^2^)	||Hi-Hm||Variance (nT^2^)
1	139.6968	3.3412 × 10^−4^	75,808	0.0790
2	274.6922	3.1591 × 10^−4^	81,159	0.0804
3	940.3178	3.1430 × 10^−4^	35,727	0.0834
4	93.0146	2.5387 × 10^−4^	115,770	0.0873

**Table 7:** Calibration results of the proposed method and traditional rotational calibration method.

Indicator	In Situ Calibration Method	Traditional Scalar Method
C_T_ Mean Value (nT/m)	3.7128	60.8081
C_T_ Variance (nT^2^/m^2^)	4.5997	349.3569

## Data Availability

The original contributions presented in this study are included in the article material. Further inquiries can be directed to the corresponding author(s).

## Abbreviations

The following abbreviations are used in this manuscript:

MGT	Magnetic gradient tensor
UXO	Unexploded ordnance
MEG	Magnetoencephalography
OPM	Optically pumped magnetometer
LM	Levenberg–Marquardt
EMC	Electromagnetic compatibility
PSO	Particle Swarm Optimization
ML	Machine learning
